# Gonadotropin releasing hormone (GnRH): a hormone therapy boosts cognition in Down syndrome and dementia

**DOI:** 10.1038/s41392-023-01321-x

**Published:** 2023-02-01

**Authors:** Keenan Sterling, Ruixue Cao, Weihong Song

**Affiliations:** 1 Townsend Family Laboratories, Department of Psychiatry, The University of British Columbia, 2255 Wesbrook Mall, Vancouver, BC Canada; 2 The Second Affiliated Hospital and Yuying Children’s Hospital, Institute of Aging, Key Laboratory of Alzheimer’s Disease of Zhejiang Province, Wenzhou Medical University, Wenzhou, China; 3 Oujiang Laboratory (Zhejiang Lab for Regenerative Medicine, Vision and Brain Health), Wenzhou, China

**Keywords:** Neurodevelopmental disorders, Molecular biology

A recent study published in *Science* by Manfredi-Lozano and colleagues suggests that the gonadotropin-releasing hormone (GnRH) could potentially restore cognition in patients with Down syndrome (DS).^1^ The findings reveal a new molecular link between dementia and the loss of smell, and a promising therapeutic avenue to combat age-related cognitive decline in DS and other neurodegenerative diseases.

DS is the most common genetic cause of intellectual and developmental disabilities. DS is typically caused by an extra copy of human chromosome 21 (HSA21). Lifelong overexpression of the HSA21-linked genes produces global alterations in gene expression patterns, resulting in a wide array of pathological and clinical manifestations. A notable feature of DS is the striking propensity for those affected to develop Alzheimer’s Disease (AD) and dementia.^2^ People with DS universally develop the histopathological marks of AD by age 40, and three-quarters of these individuals develop dementia by age 60. To date, there is no effective treatment for the cognitive symptoms, and all DS individuals experience cognitive decline as the disease progresses.

**Fig. 1:**
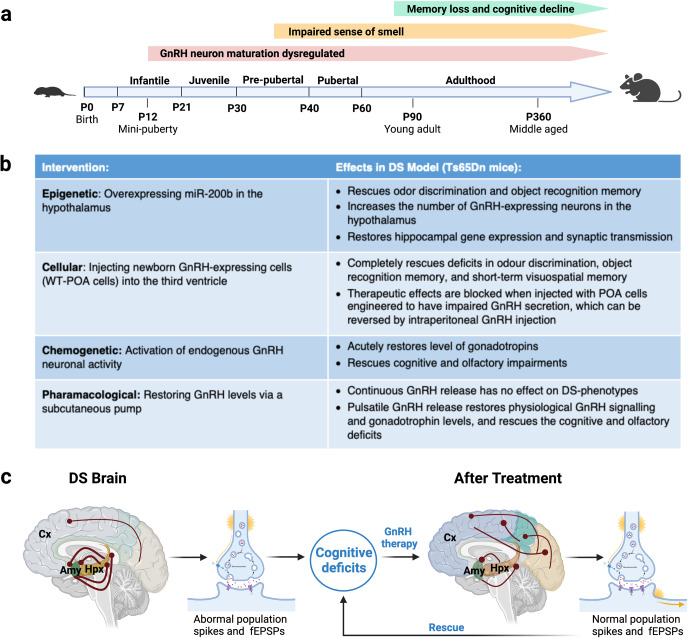
The therapeutic effects of GnRH in DS. **a** Developmental timeline showing the onset and progression of pathology in Ts65Dn mice, as reported in the highlighted paper. **b** Summary of the intervention strategies to rescue GnRH and their therapeutic effects in Ts65Dn mice. **c** Diagram showing how GnRH could boost cognition in humans. GnRH therapy may restore network dysfunction in the brain by strengthening the connectivity between cortical areas (Cx) that are hypoconnected in DS, and weakening abnormally hyperconnected subcortical areas, such as the hippocampus (Hpx) and amygdala (Amy). GnRH may also act to improve information processing at the cellular level by modulating neuronal excitability. Image created with BioRender.com. WT-POA wild-type pre-optic area, fEPSPs functional excitatory postsynaptic potentials

Another prevalent DS-associated phenotype suggests that puberty may mark a critical period in the disease progression, as many people with DS experience a progressive loss of smell and a worsening of cognitive symptoms around this time.^3^ Focusing on this subset of DS individuals, Manfredi-Lozano and colleagues identified GnRH as a potential therapeutic target by connecting four pieces of evidence. First, a genetic deficiency of GnRH results in an impaired sense of smell and delays in sexual maturity.^4^ Second, worsening olfactory symptoms coincide with cognitive decline in DS.^3^ Third, a developmental switch elevates GnRH levels just before puberty.^3^ Fourth, a small population of GnRH neurons project to the cortex and hippocampus, regions of the brain that are critical for memory and cognition and have been implicated in the pathology of dementia.^1,5^ Together, these findings demonstrate that GnRH signaling can be phenotypically, temporally, and spatially correlated with olfactory and cognitive deficits in DS, suggesting GnRH abnormalities may be causally linked to these disease phenotypes. To confirm their hypothesis, the authors characterized the progression of olfactory and cognitive symptoms in a DS mouse model (Ts65Dn mice) and assessed whether they were temporally correlated with any shifts in GnRH signaling.^1^ Similar to the human DS phenotype, olfactory deficits in Ts65Dn mice first arose during the prepubertal period and were followed by impaired memory early in adulthood (Fig. 1a). As predicted, these symptoms closely paralleled a post-pubertal loss of GnRH neuronal fibers and receptors in the hippocampus and cortex of these mice.

To determine whether GnRH signaling could serve as a viable therapeutic target, Manfredi-Lozano et al. then examined whether the cognitive symptoms could be reversed in adulthood. The team used several intervention strategies that targeted GnRH at multiple points along the proposed pathway (Fig. 1b). Notably, overexpressing a transcriptional regulator of the GnRH developmental switch (called miR-200b) in the hypothalamus of Ts65Dn mice increased the number of GnRH-expressing neurons, rectified the changes in hippocampal gene expression and neuronal activity, and rescued the olfactory and cognitive phenotypes.^1^ miR-200b can activate multiple transcriptional targets, meaning other effector pathways could underlie these changes in hippocampal gene expression and DS phenotypes. Nevertheless, every effort to restore the physiological expression and signaling patterns of GnRH successfully rescued the olfactory and cognitive deficits in Ts65Dn mice.^1^ Based on these findings, the authors conducted a pilot study to assess the effects of GnRH therapy on seven adult men with DS and olfactory deficits. The treatment involved administering regular pulses of GnRH for six months via a subcutaneous mini pump. GnRH therapy enhanced cognitive function in six of the seven DS participants, who showed significantly improved scores in higher-order executive functions, and a positive trend toward improved episodic memory.^1^ Perhaps more importantly, differences in brain imaging before and after treatment suggested that GnRH therapy may be able to alleviate cognitive impairments in DS by strengthening the communication between certain regions of the cortex while rescuing the overactivation of subcortical networks linked to the hippocampus (Fig. 1c).

The unexpected finding that GnRH therapy could have a cognitive-boosting effect in DS is very promising, with broad potential beyond DS. For example, the authors also showed that GnRH therapy could improve cognitive deficits in a mouse model of AD.^1^ The link to AD is particularly relevant considering the recent discovery that most GnRH neurons in the human basal forebrain are cholinergic,^5^ a neuronal population that is particularly vulnerable to neurodegeneration in AD. Moreover, many GnRH receptors linked to cognition also appear to be expressed in cholinergic interneurons, suggesting GnRH may exert its therapeutic effects via two possible mechanisms. First, GnRH may act locally, modulating the activity of cholinergic neurons and helping them maintain cellular parameters (i.e., synaptic transmission). Second, GnRH may modulate inhibitory relay circuits between cortical and subcortical regions of the brain, helping to maintain the functional connectivity between these brain regions. Collectively, these findings suggest that GnRH dysfunction may also play a role in the progression of neurodegenerative diseases such as AD. Moreover, GnRH therapy may have a synergistic effect with cholinesterase inhibitors, which have been shown to help alleviate the cognitive symptoms of the disease.

There is a desperate need for new therapeutic avenues to treat dementia. Pulsatile GnRH therapy is intended to mimic the natural release patterns of this hormone, making it a particularly attractive therapeutic prospect that can help to minimize any adverse side effects of long-term hormone therapy use. Caution must be urged, however. The link with human cognitive impairment is still hypothetical, and the use of GnRH therapy as a treatment for dementia would depend on whether GnRH neurons are adversely affected by aging or other pathological drivers of disease, such as neuritic plaques in AD. The study also has small clinical sample size without placebo and some more adequate cognitive assessment. In addition, the dose, course, and side effects of the treatment require further study. Nevertheless, the study by Manfredi-Lozano and colleagues employed a tour de force of experimental techniques that strongly supported the theory that GnRH dysfunction may serve as a molecular link between cognitive and olfactory deficits in DS and dementia.
